# Genetic and cultural adaptations underlie the establishment of dairy pastoralism in the Tibetan Plateau

**DOI:** 10.1186/s12915-023-01707-x

**Published:** 2023-10-05

**Authors:** Min-Sheng Peng, Yan-Hu Liu, Quan-Kuan Shen, Xiao-Hua Zhang, Jiajia Dong, Jin-Xiu Li, Hui Zhao, Hui Zhang, Xiaoming Zhang, Yaoxi He, Hong Shi, Chaoying Cui, Ouzhuluobu, Tian-Yi Wu, Shi-Ming Liu, Gonggalanzi, Baimakangzhuo, Caijuan Bai, Duojizhuoma, Ti Liu, Shan-Shan Dai, Robert W. Murphy, Xue-Bin Qi, Guanghui Dong, Bing Su, Ya-Ping Zhang

**Affiliations:** 1 State Key Laboratory of Genetic Resources and Evolution, Kunming Institute of Zoology, Chinese Academy of Sciences, Kunming, 650223 China; 2 Yunnan Key Laboratory of Molecular Biology of Domestic Animals, Kunming Institute of Zoology, Chinese Academy of Sciences, Kunming, 650223 China; 3 KIZ-CUHK Joint Laboratory of Bioresources and Molecular Research in Common Diseases, Kunming Institute of Zoology, Chinese Academy of Sciences, Kunming, 650223 China; 4 University of Chinese Academy of Sciences, Beijing, 100049 China; 5 State Key Laboratory for Conservation and Utilization of Bio-Resources, Yunnan University, Kunming, 650091 China; 6 Institute of Medical Biology, Chinese Academy of Medical Science, Peking Union Medical College, Kunming, 650118 China; 7 Key Laboratory of Western China’s Environmental Systems (Ministry of Education), College of Earth and Environmental Sciences, Lanzhou University, Lanzhou, 730000 China; 8 State Key Laboratory of Primate Biomedical Research (LPBR), School of Primate Translational Medicine, Kunming University of Science and Technology (KUST), Kunming, 650000 China; 9 High Altitude Medical Research Center, School of Medicine, Tibetan University, Lhasa, 850000 China; 10 National Key Laboratory of High Altitude Medicine, High Altitude Medical Research Institute, Xining, 810000 China; 11 The First People’s Hospital of Gansu Province, Lanzhou, 730000 China; 12 Centre for Biodiversity and Conservation Biology, Royal Ontario Museum, Toronto, ON M5S 2C6 Canada; 13 Tibetan Fukang Hospital, Lhasa, 850000 China

**Keywords:** Lactase persistence, Tibetan, Dog, Selection, Admixture, Pastoralism

## Abstract

**Background:**

Domestication and introduction of dairy animals facilitated the permanent human occupation of the Tibetan Plateau. Yet the history of dairy pastoralism in the Tibetan Plateau remains poorly understood. Little is known how Tibetans adapted to milk and dairy products.

**Results:**

We integrated archeological evidence and genetic analysis to show the picture that the dairy ruminants, together with dogs, were introduced from West Eurasia into the Tibetan Plateau since ~ 3600 years ago. The genetic admixture between the exotic and indigenous dogs enriched the candidate lactase persistence (LP) allele *10974A* > *G* of West Eurasian origin in Tibetan dogs. In vitro experiments demonstrate that − *13838G* > *A* functions as a LP allele in Tibetans. Unlike multiple LP alleles presenting selective signatures in West Eurasians and South Asians, the de novo origin of Tibetan-specific LP allele − *13838G* > *A* with low frequency (~ 6–7%) and absence of selection corresponds − *13910C* > *T* in pastoralists across eastern Eurasia steppe.

**Conclusions:**

Results depict a novel scenario of genetic and cultural adaptations to diet and expand current understanding of the establishment of dairy pastoralism in the Tibetan Plateau.

**Supplementary Information:**

The online version contains supplementary material available at 10.1186/s12915-023-01707-x.

## Background

The introduction of dairy pastoralism into East Eurasia made significant impacts on the evolution of civilization [1–5] as well as the diversity of food culture [6]. In the Tibetan Plateau, indigenous Tibetans have a long tradition of dairying domesticated yak (*Bos grunniens*), cattle, dzomo (hybrid between yak and cattle), sheep, and goat and this traces back to 4000–3000 years before present (BP) [7–9]. The analysis of ancient dental calculus from 40 human individuals from 15 sites across the Tibetan Plateau dated the dairy consumption back to at least 3500 years BP [10]. The dairy fats of pottery samples were identified at the Gongtang site (ca. 3211–2916 BP) located in the Tibet Autonomous Region of China [11]. Tibetans have developed their specific milking skills and milk processing technologies and received nutritional benefits (protein and fat) from dairy products [6]. Consequently, the introduction and development of dairy pastoralism [10, 11] together with qingke barley (*Hordeum vulgare L.*) agriculture [12] represent the cultural adaptation to the harsh environments with rough terrain, cold temperatures, and a relatively low biological productivity, which likely plays fundamental roles in permanent human occupation of the high-altitude Tibetan Plateau [13]. Nevertheless, the rise and spread of dairy pastoralism in the Tibetan Plateau remains opaque.

As an adaptation to diet shift caused by the rise of dairy pastoralism [14], lactase persistence (LP) provides a sustained ability to digest lactose contained in milk and dairy products in human adults [15]. Genetic differences in a *cis*-acting lactase gene (*LCT*) [16] constitute a heritable autosomal dominant condition [15]. Population genetic analyses and functional experiments have identified five [− *13910C* > *T* (rs4988235), − *13915 T* > *G* (rs41380347), − *13907C* > *G* (rs41525747), − *14009 T* > *G* (rs869051967), and − *14010G* > *C* (rs145946881)] (reviewed in [17, 18]) or more independent [e.g., − *14011C* > *T* (rs4988233)] single-nucleotide polymorphisms (SNPs) located around 14 kb upstream from *LCT* within intron 13 of the adjacent minichromosome maintenance 6 gene (*MCM6*) for LP [18, 19]. These LP alleles have emerged independently in several geographic/ethnic groups and have uneven distributions worldwide [17, 18]. In addition to providing a paradigm for parallel evolution [20], the LP alleles serve as informative markers in tracing the spread of dairy pastoralism and subsequent genetic admixture [21–24]. The breath hydrogen testing reveals that the proportion of lactose tolerance in Tibetans (9/30) is significantly higher than in the Han population (1/30) [25], suggesting LP may exist in Tibetans. Sequencing of the candidate enhancer region of *LCT* within *MCM6* detected SNP − *13838G* > *A* (rs1575359915 located in chr2: 136,608,574 of GRCh37) with a frequency of ~ 6.6% in Tibetans; this candidate LP allele was suggested to have had an independent origin [26]. It is still unknown if − 13838*A under selection or functionally contributes to LP in Tibetans.

Dog as commensal animal has been involved in various human migrations over time [27–30]. The dairy diaspora was accompanied with dairying ruminants (e.g., cattle, goat, and sheep) and dogs potentially for herding [31]. In particular, the A-to-G mutation at intron 2 of dog *LCT* (position chr19: 38,609,592 of Canfam3.1), named as *10974A* > *G* hereafter, has been identified as LP allele with regulatory function of increasing lactase expression in European dog breeds. Both LP alleles were positively selected in humans (− *13910C* > *T*) and dogs (*10974A* > *G*) in Europe, suggesting convergent adaptation to milk-based diets [32]. All above raise the scenario that dogs bridge pastoralists and dairy pastoralism. In the Tibetan Plateau, the indigenous dogs play indispensable roles (e.g., guard and shepherd dogs) in dairy pastoralism [33]. In addition to presenting selection signatures for high-altitude adaptation [34–37], the modern indigenous Tibetan dogs maintain unique genetic components distinguished from other dogs [38, 39]. Nevertheless, the history for dogs likely accompanying and even assisting in human occupation of the Tibetan Plateau remains obscure.

To gain insights into the history of dairy pastoralism in the Tibetan Plateau, we screened the archaeological data from 447 sites across eastern Eurasia (Additional file 1: Table S1) to show the coexistence of dairy ruminants and dogs. The demographic inference indicated Tibetan dogs receiving gene flow from West Eurasian dogs, mirroring the introduction of dairy pastoralism. Meanwhile, we leveraged multiple genetic approaches to explore the candidate functional LP allele in Tibetans and present a new example of regulatory evolution in humans. Together, we depict a picture about the gene-cultural co-evolution for the milk revolution in the Tibet Plateau.

## Results

### Geographical-temporal pattern of dairy practice and pastoralism

**Fig. 1:**
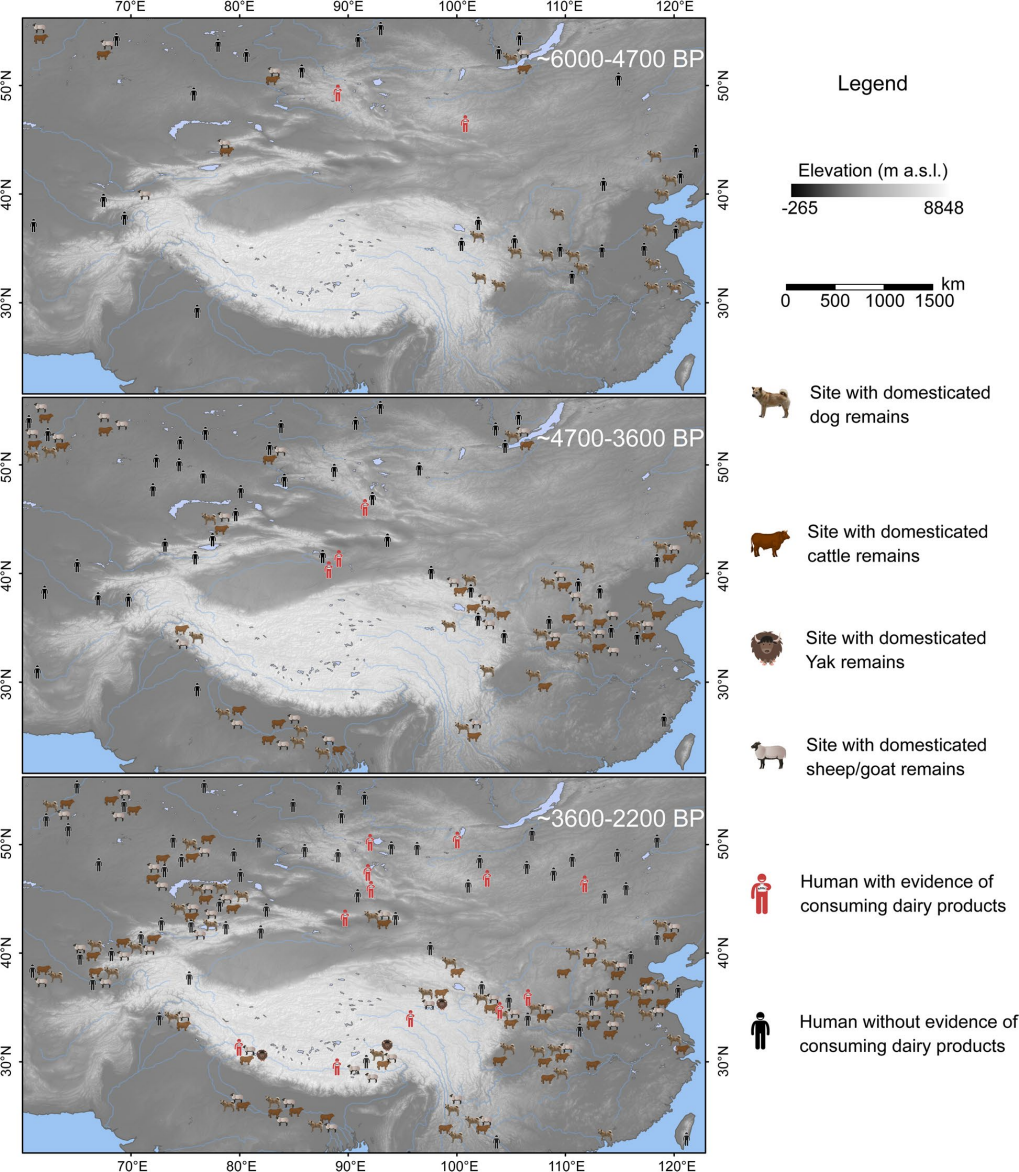
The origin and spread of pastoralism and dairying in the Tibetan Plateau. Archaeological evidence for dogs, dairy ruminants, and dairying practices in the Tibetan Plateau and the neighboring regions. The geographic mapping for each site is referred to three phases: ~ 6000–4700 BP, ~ 4700–3600 BP, and ~ 3600–2200 BP. Some sites spanning two or even three phases are mapped accordingly. Details are reported in Additional file 1: Table S1

The archaeological evidence (Additional file 1: Table S1) shows that the introduction of pastoralism based on yak, cattle, and sheep/goat into the Tibetan Plateau occurred during the period of 4700–3600 BP (Fig. 1). Nevertheless, we found no evidence indicating dairy products consumption in the Tibetan Plateau during that period, although dairy practice spread across northwestern China and Mongolia during 6000–3600 BP. The dairy products consumption was reported within ~ 3500 BP [10, 11]. During the period of 6000–4700 BP, dogs were accompanied with humans in the eastern Tibetan Plateau before the arising of pastoralism. Since 4700 BP, dogs coexisted with the ruminants on the margins (e.g., Vale of Kashmir and Hexi Corridor) of the Tibetan Plateau. After 3600 BP, the coexistence of dogs and dairy ruminants spread across the plateau. The patterns suggested dogs likely shifted the roles from food or hunting activity [40, 41] to herding assistance. How the demographic changes for dogs is unclear. The investigation of genomic data can test the proposed scenarios that the Tibetan dog population was as continuous as the indigenous dog of the Tibetan Plateau or instead admixture or even turnover occurred, possibly in relation to the influx of dairy pastoralism from West Eurasia.

### Demographic history for Tibetan dogs

**Fig. 2:**
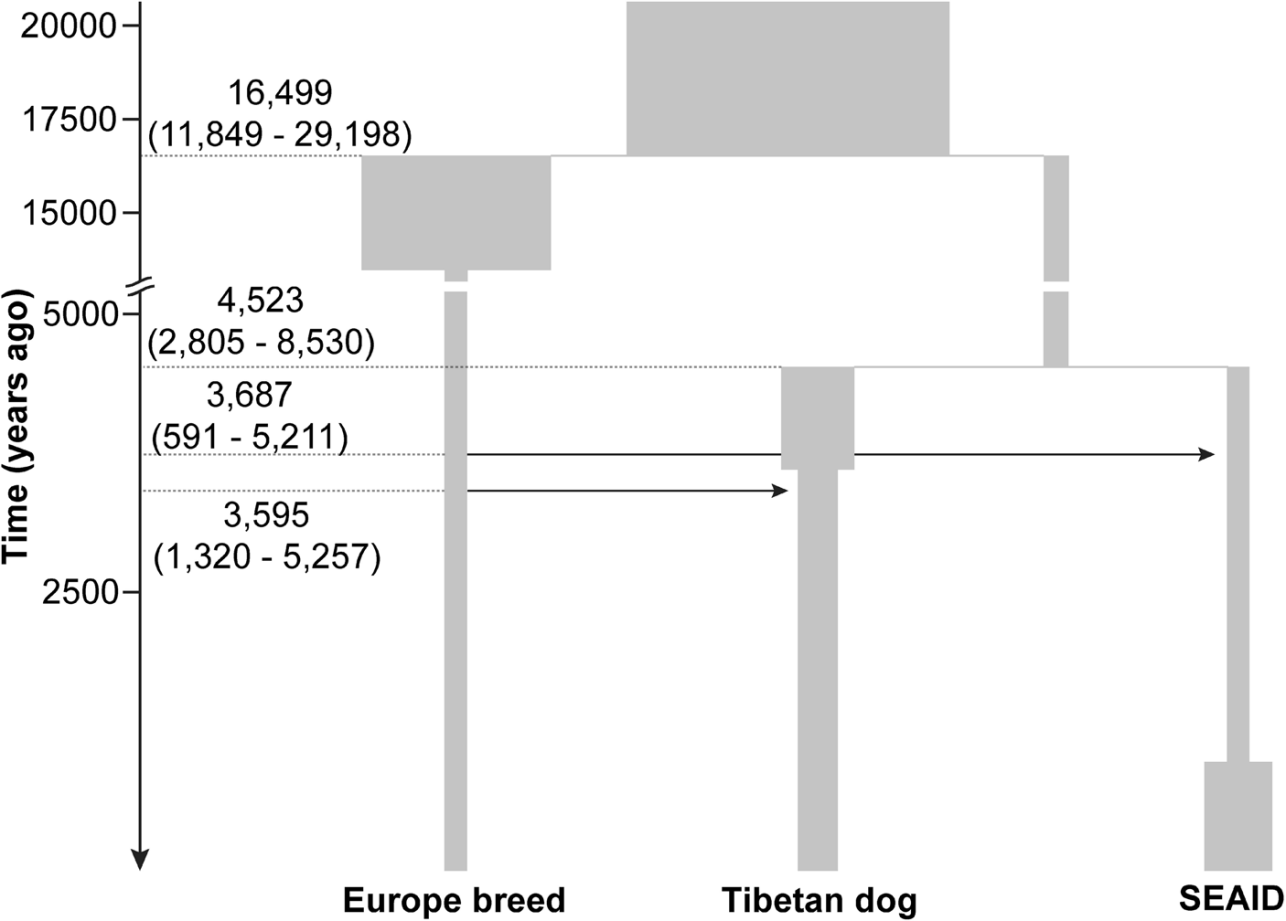
The inferred demographic history of Tibetan dogs. The time for divergence and gene flow inferred by momi2 is indicated. Estimation for other parameters is shown in Additional file 2: Fig. S4

We investigated the possibility that the spread of dairy pastoralism from West Eurasia involved the dispersal of dogs. Genomic admixture history in the Tibetan dogs was explored with referring to European dog breeds and southern East Asian indigenous dogs (SEAID) (Additional file 2: Table S2). The principal component analysis (PCA) and ADMIXTURE results revealed that modern Tibetan dogs had a closer genetic affiliation with SEAID than European breeds (Additional file 2: Figs. S1 and S2). The *D* (Andean fox, European breeds; Tibetan dogs, SEAID) statistics detected gene flow between European breeds and Tibetan dogs (*D* =  − 0.023; *Z* =  − 17.42). The local ancestral inference assigned that, on average, 30.96% of Tibetan dog genomes were derived from European breeds (Additional file 2: Table S3). Demographic modeling indicated that the model with two pulses of gene flow from European breeds into SEAID and Tibetan dogs, separately, was better than the model with only one pulse from European breeds into Tibetan dogs and the null model without gene flow (Additional file 2: Fig. S3). After Tibetan dogs split from SEAID 4523 (95% CI: 8530–2805) BP, gene flow occurred around 3595 (95% CI: 5257–1320) and 3687 (95% CI: 5211–591) BP from European breeds into Tibetan dogs and SEAID, respectively (Fig. 2; Additional file 2: Fig. S4). Gene flow between European and Tibetan dogs dated to about 4669 BP (3rd quartile 5438; 1st quartile 3900) (Additional file 2: Fig. S5) according to the criteria of 50% relative cross-coalescence rate in MSMC-IM [42]. This range overlapped with the timescale inferred by momi2 (Fig. 2; Additional file 2: Fig. S4). Consequently, the results suggested that the contacts between the ancient sources represented by European dog breeds and the Tibetan dogs occurred around 4700–3600 BP, likely before the dispersal across the Tibetan Plateau.

### Evolution of candidate LP alleles in Tibetans and their dogs

Both LP alleles were positively selected in humans (− *13910C* > *T*) and dogs (*10974A* > *G*) in Europe, suggesting convergent adaptation to milk-based diets [32]. Given that dogs likely accompany and even assist in permanent human occupation of the Tibetan Plateau [34], we focused on the candidate LP alleles − *13838G* > *A* in Tibetans and *10974A* > *G* in their dogs to assess possible convergent evolution on LP corresponding to the introduction of dairy pastoralism. Analyses of the 41 published Tibetan genomes [43, 44] detected six individuals with genotype − *13838G* > *A* as heterozygotes (allele frequency 7.3%; 6/82). In the genotyped ancient genomes from the Tibetan Plateau [45–48], the allele frequency of − *13838G* > *A* is low: 6/76 (~ 7.9%) in Nepal, 4/126 (~ 3.2%) in Tibet and 0/52 in Qinghai of China (Additional file 3: Table S4). All the samples with − *13838G* > *A* were dated no more than 2800 BP. We screened for − *13838G* > *A* in the *PGG*.SNV database [49] and detected the allele in the Balti (allele frequency 1/36) [50], Sherpa (2/10) [43], and Tu (1/4) [51] populations. The ancient and modern populations with − *13838G* > *A* have close genetic relationships with Tibetans [46, 47, 52]. It is absent in other Sino-Tibetan speakers (e.g., Han, Yi, and Naxi), raising the possibility that − *13838G* > *A* originated before the divergence of Tibetans and Sherpas but after the split of Tibetans and Han populations. The screening of *10974A* > *G* in published genomes of Eurasian dogs [53] showed that it reached a high frequency (88.6%) in 22 Tibetan dogs, especially as compared with southern East Asian indigenous dogs (SEAID, 61.8%). The frequency peaked in European breeds (91.7%). Thus, as compared with the close lowland populations, Tibetans and their dogs harbor a higher frequency of candidate LP alleles.

**Fig. 3:**
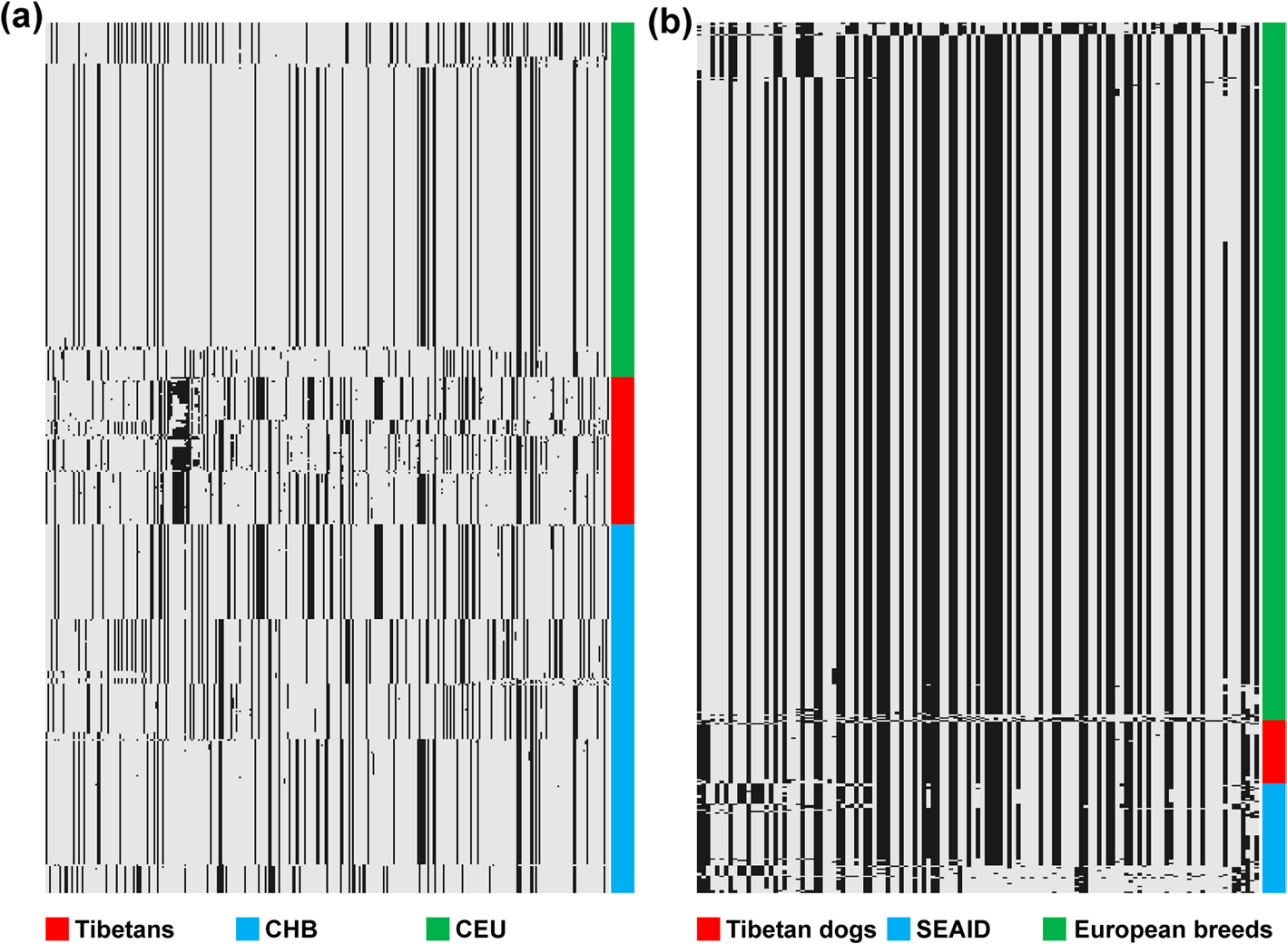
Haplotype pattern in the genomic regions with LP alleles in human and dog populations. **a** The haplotype blocks (chr2: 136,569,848–136,673,605 of GRCh37) for Tibetan, CHB, and CEU populations. **b** The haplotype blocks (chr19: 38,607,365–38,659,515 of Canfam3.1) for Tibetan dog, southern East Asian indigenous dogs, and European breeds. Each column is a polymorphic genomic location, each row is a phased haplotype from human or dog populations noted with different colors

To trace the origin of LP alleles in Tibetans and their dogs, we compared the core haplotypes containing LP alleles in the genomes of Tibetans and their dogs with the Eurasian populations and dog breeds, respectively. The Tibetan and Han (Han Chinese in Beijing, CHB) harbored similar haplotypes (chr2: 136,569,848 − 136,673,605 of GRCh37), but they were distinct from those in European population (Utah residents with Northern and Western European ancestry from the CEPH collection, CEU) suggesting a de novo origin of − *13838G* > *A* derived from Asian haplotype background (Fig. 3a). In contrast, the haplotype blocks (chr19: 38,607,365–38,659,515 of Canfam3.1) of Tibetan dogs are almost identical to those of European breeds (Fig. 3b). Around 70.5% haplotypes with *10974A* > *G* in Tibetan dogs were inferred to be from European breeds. Additional sliding-window *D* and *f*_*dM*_ statistics listed *lct* including *10974A* > *G* with positive scores (*D* = 0.483 and *f*_*dM*_ = 0.559) as the top 1% of genomic regions (Additional file 2: Fig. S6). These results indicated that the LP allele *10974A* > *G* was likely derived via gene flow from European breeds into Tibetan dogs.

**Fig. 4:**
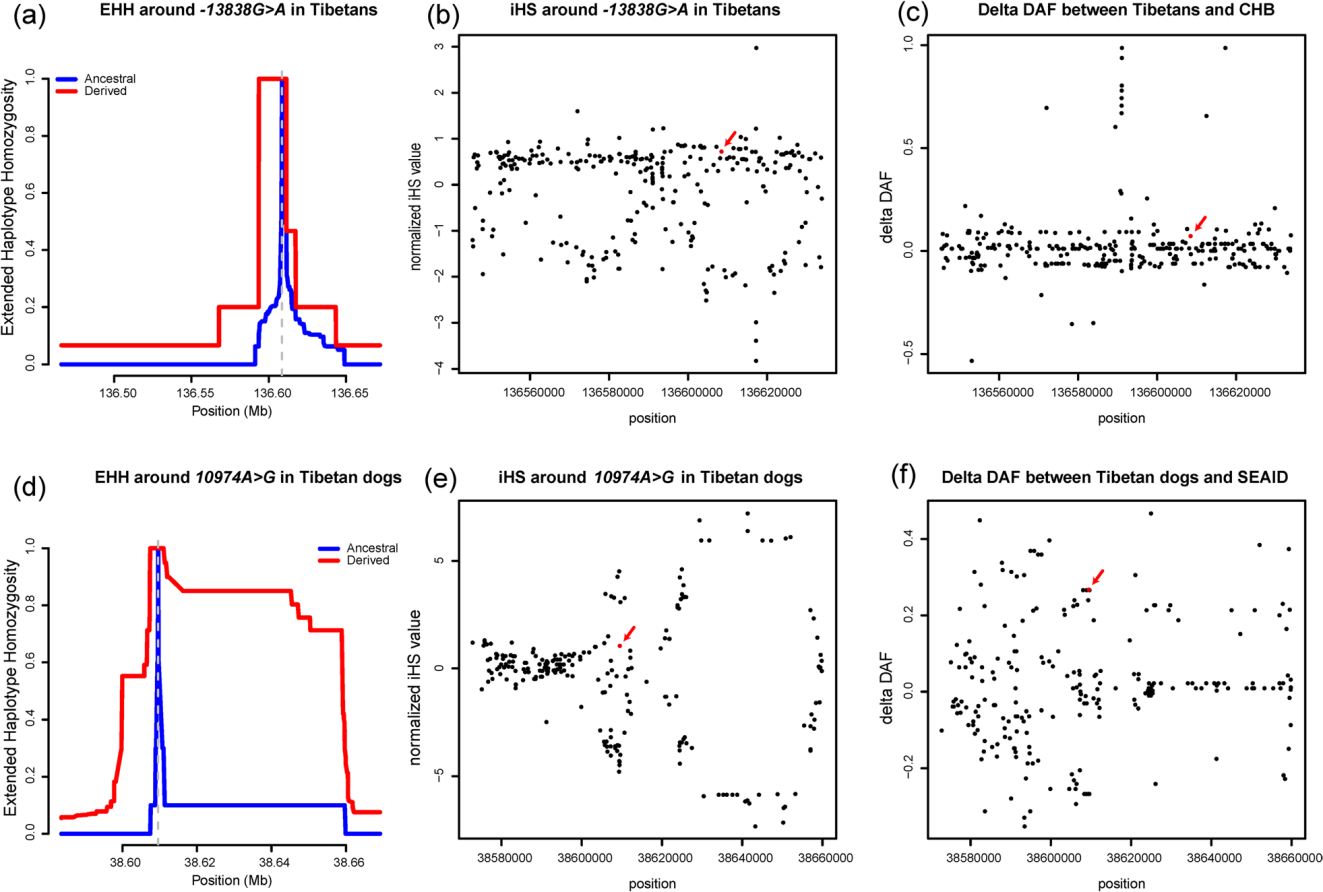
Tests of selective sweep on LP alleles in Tibetans and their dogs. **a** EHH of the derived − 13838*A haplotypes (in red) in comparison with the ancestral − 13838*G haplotypes (in blue). **b** iHS for − *13838G* > *A* (in red) and its surrounding region. **c** The delta DAF for the region around − *13838G* > *A* between the Tibetan and Han Chinese populations. **d** EHH of the derived 10974*G haplotypes (in red) in comparison with the ancestral 10974*A haplotypes (in blue). **e** iHS for *10974A* > *G* (in red) and its surrounding region. **f** The delta DAF for the region around *10974A* > *G* between the Tibetan dogs and southern East Asian indigenous dogs

We further tested the potential selection on the candidate LP alleles − 13838*A in Tibetans and 10974*G in their dogs. Phased haplotype data showed evidence of extended haplotype homozygosity (EHH) [54] when − 13838*A occurred as compared with haplotypes with ancestral − 13818*G (Fig. 4). The integrated haplotype score (iHS) for − 13838*G was 1.70, less than the statistically significant threshold of 2. The screening of derived allele frequency change (delta DAF) [55] between the Tibetan and CHB populations presented no evidence of selection on − *13838G* > *A*. Similar patterns were observed in the EHH, iHS, and delta DAF tests for *10974A* > *G* in Tibetan dogs (Fig. 4). The results did not provide evidence of selection on the candidate LP alleles in both Tibetans and their dogs.

### Functional investigation of Tibetan − *13838G > A*

**Fig. 5:**
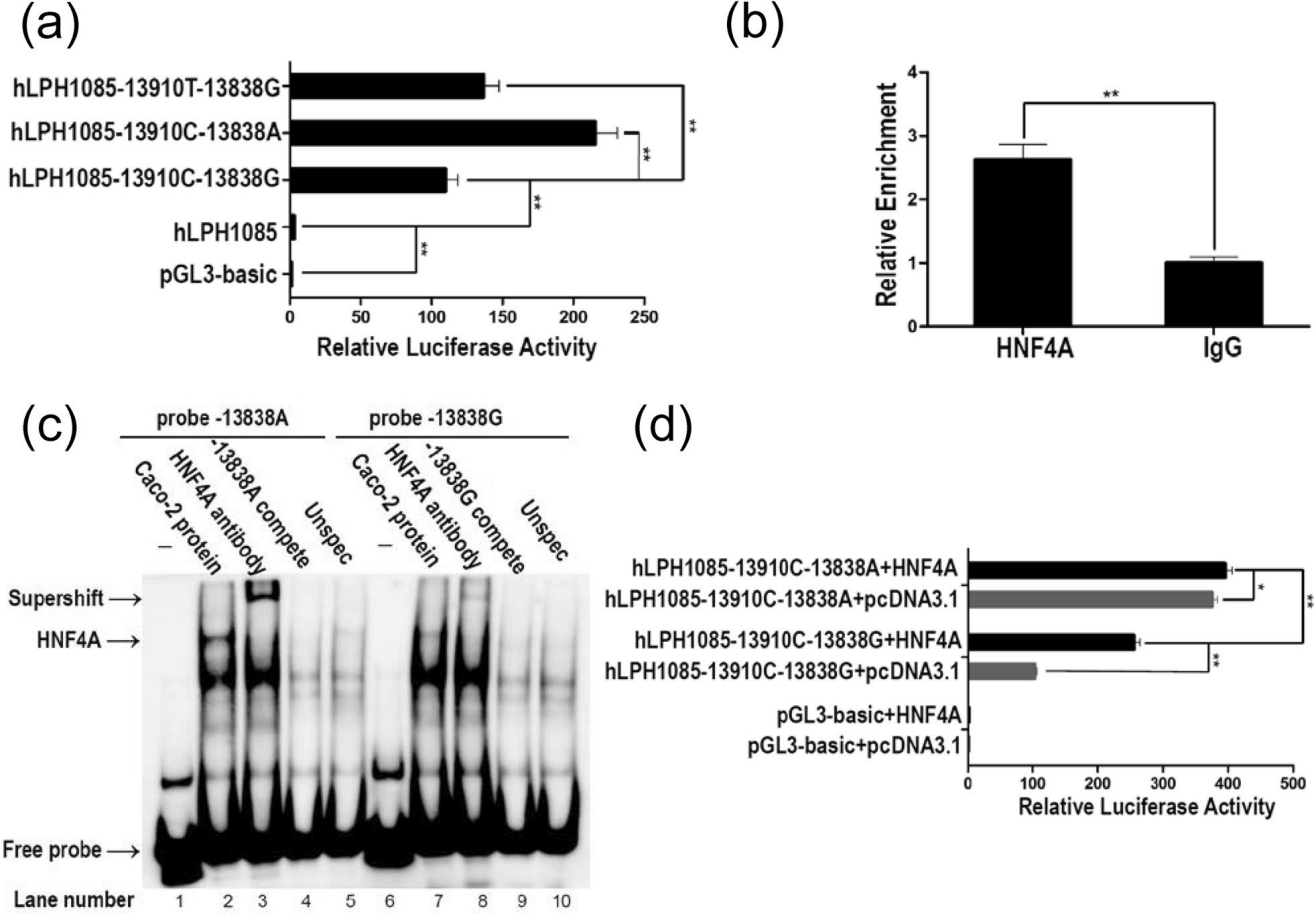
Functional analysis of − *13838G* > *A* as enhancer in Caco-2 cells. **a** The luciferase reporter assays of *LCT* promoter and enhancer constructs. As a control, cells were transfected with the promoterless pGL3-basic vector. Basal levels of expression were assessed using hLPH1085 constructed with a pGL3-basic vector and 1085 bp *LCT* promoter. Three different enhancer haplotypes were inserted upstream of the hLPH1085. Differences in expression of the reporter gene were indicated by the relative luciferase activities. **b** ChIP-qPCR for the DNA–protein complex enriched by anti-HNF4A antibody. The complex enriched by anti-IgG antibody was amplified as the control. **c** Electrophoretic mobility shift assays with Caco-2 cell nuclear extracts and oligonucleotide probes spanning − *13838G* > *A* SNP. Specific competitors: − 13838*A (lane 4) or − 13838*G (lane 9) probes without biotinylation; unspecific competitor: unlabeled oligonucleotide probe in which the 8 bp nucleotides surrounding − *13838G* > *A* were mutated (lanes 5 and 10). **d** The luciferase reporter assays for caco-2 cells co-transfected with the *LCT* promoter and enhancer constructs harboring − 13838*A or − 13838*G in the presence of HNF4A (hLPH1085 − 13910C − 13838A/G + HNF4A) and pcDNA3.1 expression constructs (hLPH1085 − 13910C − 13838A/G + pcDNA3.1). Statistical significance was tested using the unpaired Student’s *t*-test: *0.01 < *P* < 0.05; **P < 0.01

We used luciferase report assays to test for transcription activity of − *13838G* > *A*, in which − *13910C* > *T* served as the positive control [56]. Enhancers containing either derived variant − 13838*A or − 13910*T (as positive control) had significantly higher relative luciferase activity (*P* < 0.01) than ancestral variants − 13838*G and − 13910*C (Fig. 5a). ChIP-qPCR indicated that HNF4A was capable of interacting with the − *13838G* > *A* region (Fig. 5b), as revealed by screening the JASPAR database and previous *DNase I* footprint experiments [57]. EMSAs (Fig. 5c) showed that HNF4A protein can bind the − *13838G* > *A* region (lanes 3 and 8). And − 13838*A (lanes 3, super-shift band) was more capable of binding to HNF4A than − 13838*G (lanes 8). The co-transfections with the HNF4A (Fig. 5d) showed that − 13838*A presented an enhanced regulatory effect as compared with − 13838*G (*P* < 0.01). Thus, − 13838*A acts as an enhancer and binds to HNF4A to up-regulate the expression of *LCT* in vitro.

To check the functional role of − *13838G* > *A* in vivo, we genotyped − *13838G* > *A* and screened for the LP phenotype in a cohort of 32 adult Tibetans (Additional file 2: Table S5). Two individuals were heterozygote carriers of − *13838G* > *A*. The hydrogen breath tests showed two individuals had the LP phenotype. One individual carried − *13838G* > *A* in a heterozygote. The low frequency of LP and − *13838G* > *A* and the small sample size precluded a statistical association analysis of phenotype and genotype.

## Discussion

Reconstructing the history of human activities on the Tibetan Plateau is crucial for understanding adaptation to high-altitude environments [13]. A series of studies revealed mechanisms for genetic and physiological adaptation to hypoxia in Tibetans [58–60] and their domestic animals [61–63]. Herein, we provide a novel perspective of cultural adaptation with nutritional and technological advantages based on secondary products from dairy livestock to the harsh “roof of the world.” The integration of archaeological (Fig. 1; Additional file 1: Table S1) and genetic (Fig. 2; Additional file 2: Figs. S4 and S5) evidence reveals West Eurasian dogs have been involved in the introduction of dairy pastoralism into East Eurasia via the trans-Eurasia exchange during the Late Neolithic and Bronze Age [1, 64]. After admixture with local East Eurasian dogs, some dogs (most likely used in herding) were accompanied with the dairy ruminants such as yak and sheep, contributing to permanent human occupation of the Tibetan Plateau within 4000 BP [12, 13]. The scenario is also consistent with genetic inference of demographic history for sheep [63, 65] and qingke barley [66] in the Tibetan Plateau. The ancestors of Tibetans received the cultural package of agropastoralism including dairy ruminants, dogs, and qingke barley, but very limited gene flows [43, 52, 67], from West Eurasia. Our results highlight the tight link between dogs and livestock since the Bronze Age that still continues in modern Tibetan communities practicing traditional pastoralism across the Tibetan Plateau.

Our study reveals the existence of LP allele − *13838G* > *A* that potentially functions to increase the expression of lactase gene in vitro in Tibetans (Fig. 5). Unlike other populations having a practice of dairy pastoralism in East Eurasia harboring known LP alleles [24, 68], − *13838G* > *A* in Tibetans presents a novel case for regulatory evolution of LP in human populations. However, our study has several important limitations. First, we acknowledge that the current small sample sizes of Tibetans for both whole genome-sequencing (*n* = 41) and genotype–phenotype association (*n* = 32) limit the statistical power in our data analyses, especially given that multiple factors such as lactose dosage and intestinal microbiome can affect the diagnosis of LP [69]. Second, our in vitro experiments are not sufficient to present direct evidence for − *13838G* > *A* upregulating *LCT* expression. The epigenetic analyses of biopsy samples [70–72], CRISPR/Cas9-edited intestine-derived cells [70], and building a transgenic animal model [73], can provide further insights into the functional roles − 13838*A play for LP in Tibetans.

The LP allele − *13838G* > *A* occurred at low frequency both in modern Tibetans (6–7%) [26, 74] and ancient populations (Additional file 3: Table S4) from the Tibetan Plateau as compared with LP alleles with selective sweeps in populations with milk-drinking traditions [17], e.g., − *13910 C* > *T* in northern Europeans (61.5%) and South Asians (16.7%), and − *13915 T* > *G* in Middle Eastern populations (9.4%) [18]. The selective signals detected in − *13838G* > *A* of Tibetans (Fig. 4) are much weaker than − *13910C* > *T* of Europeans [55]. The integration of multiple selective signals showed no signatures of Darwinian-positive selection in − *13838G* > *A* of Tibetans [74]. These patterns were in accordance with the results of − *13910 C* > *T* (~ 7%) in modern Mongolians without identified selective signal [68]. The West Eurasian-originated − *13910 C* > *T* maintained low frequency and was unlikely under selection since the Late Bronze Age across the eastern Eurasian steppe [24, 75, 76]. Thus, although − *13838G* > *A* of Tibetans and − *13910 C* > *T* of Mongolians or Central Asians have different origins, both LP alleles show similar patterns of low-frequency distribution and absence of selection, presenting a case of parallel evolution. It raises the possibility that culture and/or gut microbiota adaptations [24] contribute to LP in Tibetans. The development of fermentation technology by Tibetans [77], which likely accompanied the introduction of dairy pastoralism [78], substantially reduces lactose dose in milk [79] and relaxes the selective pressure driven by lactose digestion [17]. Alternatively, the calcium assimilation hypothesis proposes that LP was selectively favored to provide vitamin D and calcium to avoid rickets [80]. The strong UV irradiation in the Tibetan Plateau [81] may further relieve the potential selective pressure from poor vitamin D status [82]. Moreover, recent studies ascribed the evolution of − *13910C* > *T* in Europeans to the adaptation to famine and disease [83, 84]. To decipher the episodic evolution of − *13838G* > *A* in Tibetans, more efforts from genetics, archaeology, and medicine are still required.

## Conclusions

In summary, our study shows that integrating of high-resolution genomic data, ancient DNA, and archaeological evidence of human populations and their commensal domestic animals can update and extend our knowledge of the agropastoral economic history. We depict a picture that the introduction of West Eurasian-originated agropastoralism into East Eurasia was accompanied with dogs. The ancestors of Tibetans received the agropastoral package, hybridized the exotic dogs with their indigenous dogs, and finally spread across the Tibetan Plateau, in which the perplexing evolution of LP allele − *13838G* > *A* was likely involved. Disentangling the milk revolution in both genetic and cultural shifts in the Tibetan Plateau has proven more challenging. Given the multiple waves of migrations into or around the Tibetan Plateau over time [13, 46, 52, 85–87], the current demographic modeling of dog population history is broad-stroke with a loss of nuance. To provide more details, the analysis of a dense spatial and temporal dataset including both modern and ancient genomes is required for humans [46, 48, 88, 89] and dogs [30, 90] as well as other domestic animals [91] in the Tibetan Plateau and the surrounding regions. The investigation of ancient milk lipids, proteins, and fermentation microorganisms [4, 83, 92, 93] will also be an important direction for future work.

## Methods

### Collection of archaeological data

We collected archaeological data from 447 sites in 15 countries across eastern Eurasia ranging from around 6000–2200 BP (Additional file 1: Table S1). The Tibetan Plateau and surrounding regions were included. We screened the sites with remains from domesticated dog, cattle, yak, and sheep/goat. The evidence of consuming dairy products was also checked.

### Genomic data of dog populations

Whole genomic variation of 722 modern canines (Additional file 2: Table S2) was downloaded from NCBI [53]. Autosomal SNPs marked with PASS are used. To avoid sampling bias (from *n* = 1 to *n* = 44 per breed) in European breeds, we followed the strategy reported previously [32] to select five samples with the highest genome coverage for every breed with n > 5. We used all samples for breeds with n ≤ 5. The PCA was carried out using smartpca in EIGENSOFT (v7.2.1) [94]. The unsupervised ancestry component clustering was performed with ADMIXTURE [95]. After removing outliers identified by PCA and ADMIXTURE, we kept the genomic data for 242 European dog breeds, 38 SEAID, 22 Tibetan dogs, and 41 Gy wolves for further analysis. Based on the genetic map (https://github.com/auton1/dog_recomb) [96], canine genomic genotypes were phased by SHAPEIT v2.r904 with 0.5 Mb windows and an effective population size of 83,600 [97]. For each SNP, ancestral or derived allelic status was determined according to the genotype of Andean fox (*Lycalopex culpaeus*) [96].

### Admixture inference for Tibetan dogs

We set the Tibetan dogs as the target population and European breeds and SEAID as source populations and then used PCAdmix V1.0 [98] for local ancestry inference. The sliding-window *D* and *f*_*dM*_ statistics were calculated with “-P1 SEAID -P2 Tibetan Dogs -P3 European breeds -O Fox”, using window size 100 kb and step 20 kb [99]. Each window contained at least 100 SNPs. MSMC-IM [42] was run for four haplotypes of two dogs from European breeds and two Tibetan dogs sequenced with the highest depth. After mapping the reads to the dog reference genome Canfam3.1 with BWA-MEM [100], base quality score recalibration was conducted by using GATK [101]. SNPs were called by mpileup in samtools with parameter: “-q 20 -Q 20 -C 50” [102]. Mask files of individuals were generated using bamCaller in msmc-tools (www.github.com/stschiff/msmc-tools). Genome mapability mask file generated through the program SEQBILITY (https://github.com/lh3/misc) with referring to the protocol (http://lh3lh3.users.sourceforge.net/snpable.shtml). Coalescence rates were calculated using “-I 0,1,2,3” for Tibetan dogs and “-I 4,5,6,7” for European breeds. We used “-I 0–4,0–5,0–6,0–7,1–4,1–5,1–6,1–7,2–4,2–5,2–6,2–7,3–4,3–5,3–6,3–7” to obtain estimates of the coalescence rates across populations. We used a mutation rate 1.33e − 9 mutations per year [103].

### Modeling the demography of Tibetan dogs

Using the same dataset as in the MSMC-IM analysis above, we extracted the genome-wide site frequency spectrum (SFS) and inferred the demographic history under the phylogeny (European breeds, (Tibetan dogs, SEAID)) with momi2 [104]. The SNPable regions were inferenced using SNPable (http://lh3lh3.users.sourceforge.net/snpable.shtml). Three models allowing the change of effective population size in branches leading to the current populations were proposed (Additional file 2: Fig. S3): (1) the null model without gene flow; (2) the one pulse model introducing the gene flow from European breeds into Tibetan dogs; (3) the two pulses model containing gene flow from European breeds into Tibetan dogs and SEAID, respectively. We referred to the previously inferred population history of dogs to set the priors [105, 106]. The mutation rate per generation was set to 4.5e − 9 with the generation time of three years. The effective population size was allowed to vary between 1e3 and 5e5. The split time between European dogs and Asian dogs was constrained to between 1e4 and 3.3e4 BP. For each model, 100 independent runs with different random start seeds were performed. The distribution of loglikelihood values for each model was calculated and then used for model selection based on the *t* test (two-tailed). For the selected model, we split the SFS into 100 contiguous blocks with equal size and then bootstrapped 100 SFS blocks. We performed independent momi2 runs with the 100 bootstrapped SFS. The 100 runs were used to obtain the 95% CI for parameter estimation.

### Genomic data of human populations

We followed the standard GATK pipeline [101] to call the variants based on the published whole-genome sequencing reads of 41 Tibetan individuals [43, 44] as described before [51]. Reads were mapped to reference human genome build GRCh37 by using BWA-MEM [100]. Picard was used to mask the PCR duplicates and produced the file named with dedup.bam. Local alignment was performed to adjust the alignments via GATK indel realignment of GATK score recalibration modules. Setting known sites as training data, we re-calculated base quality via the base quality score recalibration (BQSR) module. After using the GATK HaplotypeCaller module to generate the genomic VCF (gVCF) file for each individual, we conducted joint calling of all gVCF files via the GenotypeGVCFs module and obtained a raw VCF file for 41 individuals. The variant quality score recalibration (VQSR) module of GATK was used to filter variants with low quality.

For human genomic data, we phased the Tibetan genotypic data by using Beagle 4.1 [107] without a reference panel, and the parameters were set by default. Only biallelic SNPs were retained for the subsequent analyses. To determine the ancestral and derived state, we downloaded the results generated by the 1000 Genomes Project Phase III [108], which were obtained by using the 6-way EPO alignments available in Ensembl v71.

### Analysis of LP alleles in human and dog populations

We focused on the candidate LP alleles − *13838G* > *A* in Tibetans and *10974A* > *G* in dogs. The phased haplotypes from CEU and CHB populations of the 1000 Genomes Project [108] were used to check the genetic affiliation with Tibetan haplotypes. For human genomic data, we extracted the haplotypes data covering both ~ 1 Mb in upstream and downstream surrounding − *13838G* > *A* and used the block_calculation function in R package HaploBlocker [109] to show the haplotype blocks containing the LP alleles (~ 100 kb; chr2: 136,569,848 − 136,673,605 of GRCh37). The same strategy was applied for the haplotypes (~ 50 kb; chr19: 38,607,365–38,659,515 of Canfam3.1) from European breeds, SEAID, and Tibetan dogs.

We calculated EHH [54] and iHS [110] with REHH v2.0 [111] to investigate the recent selection on LP alleles in Tibetan human and dog populations based on the phased chromosomal data with default parameters. We kept the variants with minor allele frequency more than 0.05 in the analysis. The original iHS values were *z*-score normalized using the whole chromosome data as control, which was chromosome 2 for humans and chromosome 19 for dogs, respectively. Based on the same genomic regions, we calculated the difference in delta DAF [55] between the Tibetan and CHB populations. The delta DAF between Tibetan dogs and SEAID was also evaluated.

### In vitro investigation of − *13838G > A*

We investigated the influence of the variant − 13838*A on the *LCT* enhancer using Caco-2 cells, a colon carcinoma cell line widely used in LP studies [56]. Caco-2 cells were maintained in Dulbecco’s modified Eagle’s medium (DMEM) supplemented with 10% fetal bovine serum (FBS), 100 U/ml penicillin, and 100 μg/ml streptomycin. Cells were grown in a humid environment at 5% CO_2_ and 37 ℃ and were split at 80% confluence (after 2–3 days).

We followed the reported protocol to construct plasmids [56]. We introduced − *13910C* > *T*, which has a definite enhancer function in European population [56, 112], as a positive control to study the role of − *13838G* > *A* in Tibetans. Genomic DNA was taken from a Tibetan individual previously identified with − 13838*G [26]. A Human *LCT* gene promoter of 1,085 bp fragment (nucleotide position from − 1097 bp to − 13 bp to the start codon of *LCT*) was PCR amplified and inserted into the pGL3-basic vector to construct the plasmid hLPH1085. A 450 bp fragment of enhancer (nucleotide position from − 14,133 bp to − 13,684 bp to the start codon of *LCT*) with ancestral alleles − 13838*G and − 13910*C was amplified and then cloned into the hLPH1085 to make the plasmid hLPH1085 − 13838G − 13910C. Plasmids of hLPH1085 − 13838A − 13910C and hLPH1085 − 13838G − 13910 T with derived alleles were generated using Phusion Site-Directed Mutagenesis Kit (Thermo Fisher Scientific). Sanger sequencing verified the sequences of plasmids. The plasmids were then transfected into Caco-2 cells. In the luciferase reporter assays, the cells were transfected with a total plasmid DNA amount of 0.5 µg per well including 475 ng luciferase reporter plasmid and 25 ng pRL-TK (Promega) as an internal control by 1.5uL ViaFect (Promega). After 24 h transfection, cells were harvested and tested according to the Dual-Luciferase Reporter Assay System (Promega) manufacturer’s protocol. The JASPAR database (http://jaspar.genereg.net/) [113] was used to predict the regulation of transcription factor HNF4A bounding near − *13838G* > *A*. In the co-transfections, 200 ng of HNF4A expression plasmids or pcDNA3.1 (control) were transfected along with 400 ng of the luciferase reporter plasmids. Each transfection experiment was repeated four times.

ChIP was performed by EZ-ChIP Assay Kit (EMD Millipore) according to the manufacturer’s protocol. Caco-2 cells were cross-linked by 1% formaldehyde in DMEM for 10 min at room temperature. After quenching with glycine, the cells were scraped and lysed. The chromatin complex was ultrasonicated into sizes ranging from 200 to 1000 bp and then checked using agarose gels. The prepared chromatin complex was subjected to immunoprecipitation with mouse monoclonal anti-HNF4A antibody (Santa Cruz Biotechnology), and normal mouse IgG was used as a negative control. Real-time qPCR was carried out with SYBR® premix EX-Taq (TaKaRa) on QuantStudio 5 Real-Time PCR System (Thermo Fisher Scientific) to qualify the binding of HNF4A. The qualification was normalized with the chromatin without immunoprecipitation in the real-time qPCR. The primers used in qPCR were 5′-GCAGGGCTCAAAGAACAATC-3′ and 5′-GCGCTGGCAATACAGATAAG-3′. Each real-time qPCR was performed IN three independent runs.

EMSAs were performed using a LightShift Chemiluminescent EMSA Kit following the manufacturer’s instructions (Thermo Fisher Scientific) and previous studies [56, 114]. Double-stranded oligonucleotide probes and competitors are listed in Additional file 2: Table S6. Nuclear proteins from the Caco-2 cells were extracted using NE-PER™ Nuclear and Cytoplasmic Extraction Reagents (Thermo Fisher Scientific) and then quantified with the Pierce BCA Protein Assay Kit (Thermo Fisher Scientific). For competition experiments, either specific or non-specific unlabeled competitors were pre-incubated with 9 µg of nuclear extract for 20 min at room temperature before the addition of the labeled probes. Unlabeled competitor probes were added into reaction at a 100-fold concentration as compared with labeled probes. In separate experiments to test for the presence of specific proteins within the observed complexes, the nuclear protein mixture was incubated for 40 min at room temperature with antibody HNF4A (Santa Cruz, sc-374229 X).

### Phenotype–genotype investigation in Tibetans

All procedures were in accordance with the revised Helsinki Declaration of 2000 and were approved by the Internal Review Board of Kunming Institute of Zoology, Chinese Academy of Sciences. We conducted hydrogen breath tests [115] for 32 unrelated Tibetan volunteers (aged 17–63 years) recruited from the Tibet Autonomous Region, China in 2006. The volunteers were instructed to fast overnight and avoid smoking. The baseline of breath hydrogen was measured in parts per million (ppm) using a Micro H2 Analyzer (Micro Medical Limited, Chatman, UK). A tolerance test dose of 50-g lactose powder diluted in 250 mL of water was given to each volunteer. If not being hydrolyzed by lactase, lactose will be broken down by intestinal bacteria with hydrogen as a by-product. The breath hydrogen was measured at 30-min intervals over a 3-h period. The increment in breath hydrogen value of less than 20 ppm over the baseline, suggesting lactose mainly being digested rather than processed by bacteria, was classified as LP. Genomic DNA was extracted from the blood samples of 32 unrelated Tibetan volunteers for hydrogen breath tests by using the phenol–chloroform method. The 321-bp regulatory region for *LCT* (position − 14044 to − 13724 upstream *LCT*) in intron 13 of *MCM6* was amplified and sequenced as described before [116].

### Supplementary Information

**Additional file 1: Table S1.** The information for archaeological sites.

**Additional file 2: Table S2.** Sample information for genomic data of dogs and wolves. **Table S3.** The local ancestral inference for Tibetan dogs with PCAdmix. **Table S5.** Lactose tolerance test and lactase persistence allele genotyping for 32 adult Tibetans. **Table S6.** Information of double-stranded oligonucleotide probes in EMSAs. **Fig. S1.** Principal component analysis showing genetic affiliation among Tibetan dogs, southern East Asian indigenous dogs, European breeds, and wolves. **Fig. S2.** ADMIXTURE analysis for Tibetan dogs, southern East Asian indigenous dogs, European breeds, and wolves. **Fig. S3.** Model selection by momi2. **Fig. S4.** The selected model with two pulses of gene flow and parameter estimation. **Fig. S5.** The admixture history of Tibetan dogs inferred by MSMC-IM. **Fig. S6.** Sliding window *D* and *f*_*dM*_ statistics (outgroup Andean fox, European breeds;  Tibetan dogs, southern East Asian indigenous dogs) around the dog lactase gene.

**Additional file 3: Table S4.** The genotyping of −*13838G/A* in ancient DNA samples in the Tibetan Plateau.

## Data Availability

All data generated or analyzed during this study are included in this published article, its supplementary information files, and publicly available repositories. All genomic data are published previously. The eight and 33 Tibetan genomes are available at GSA (https://ngdc.cncb.ac.cn/gsa/) with PRJCA000600 and PRJCA000246, respectively. The canine genomes are available at NCBI with access number PRJNA448733.

## Abbreviations

BP: Before present
BQSR: Base quality via base quality score recalibration
CEU: Utah residents with Northern and Western European ancestry from the CEPH collection
CHB: Han Chinese in Beijing
ChIP: Chromatin immunoprecipitation
delta DAF: Derived allele frequency change
DMEM: Dulbecco’s modified Eagle’s medium
EHH: Extended haplotype homozygosity
EMSA: Electrophoretic mobility shift assay
FBS: Fetal bovine serum
gVCF: Genomic VCF
HNF4A: Hepatocyte nuclear factor 4α
iHS: Integrated haplotype score
*LCT*: Lactase gene
LP: Lactase persistence
*MCM6*: Minichromosome maintenance 6 gene
PCA: Principal component analysis
ppm: Parts per million
SEAID: Southern East Asian indigenous dogs
SFS: Site frequency spectrum
SNPs: Single-nucleotide polymorphisms
VQSR: Variant quality score recalibration
