# Supplementary material for: Genetic and cultural adaptations underlie the establishment of dairy pastoralism in the Tibetan Plateau
Source: BMC Biol. 2023 Oct 5;21:208. doi: 10.1186/s12915-023-01707-x (PMC10557253; doi:10.1186/s12915-023-01707-x)

Additional file 2

Table S2. Sample information for genomic data of dogs and wolves.

Group	Sample ID	Breeds
	149323_S6	Labrador Retriever
	152721_S5	Labrador Retriever
	160822_S4	Labrador Retriever
	165414_S20	Rottweiler
	172384_S14	Golden Retriever
	173006_S10	Golden Retriever
	175885_S12	Golden Retriever
	91072_S13	Golden Retriever
	AiredaleTerrier01	Airedale Terrier
	AiredaleTerrier02	Airedale Terrier
	AiredaleTerrier05	Airedale Terrier
	BAN00024	Miniature Poodle
	BAN00032	Yorkshire Terrier
	BAN00127	Yorkshire Terrier
	BC0480	Border Collie
	BC518	Border Collie
	BG064	Bayerischer Gebirgsschweisshund
	BT007	Miniature Bull Terrier
	BU002	Bullmastiff
European breeds (EB)	Beagle01	Beagle
	Beagle02	Beagle
	Beagle03	Beagle
	Beagle04	Beagle
	BeardedCollie01	Bearded Collie
	BeardedCollie02	Bearded Collie
	BeardedCollie03	Bearded Collie
	BeardedCollie04	Bearded Collie
	BeardedCollie05	Bearded Collie
	BelgianMalinois01	Belgian Malinois
	BelgianMalinois02	Belgian Malinois
	BelgianMalinois03	Belgian Malinois
	BelgianSheepdog01	Belgian Sheepdog
	BelgianSheepdog03	Belgian Sheepdog
	BelgianSheepdog04	Belgian Sheepdog
	BelgianSheepdog06	Belgian Sheepdog
	BelgianSheepdog07	Belgian Sheepdog
	BelgianTervuren01	Belgian Tervuren
	BelgianTervuren05	Belgian Tervuren
	BelgianTervuren07	Belgian Tervuren
	BelgianTervuren10	Belgian Tervuren

BelgianTervuren11	Belgian Tervuren
BergerBlancSuisse01	Berger Blanc Suisse
BergerPicard01	Berger Picard
BergerPicard02	Berger Picard
BergerPicard03	Berger Picard
BerneseMountainDog01	Bernese Mountain Dog
BerneseMountainDog02	Bernese Mountain Dog
BerneseMountainDog12	Bernese Mountain Dog
BerneseMountainDog14	Bernese Mountain Dog
BerneseMountainDog18	Bernese Mountain Dog
Bloodhound01	Bloodhound
BorderCollie07	Border Collie
BorderCollie08	Border Collie
BorderTerrier01	Border Terrier
BorderTerrier02	Border Terrier
BorderTerrier03	Border Terrier
BouvierDesFlandres01	Bouvier des Flandres
BouvierDesFlandres02	Bouvier des Flandres
BrittanySpaniel01	Brittany
BullTerrier01	Bull Terrier
BullTerrier03	Bull Terrier
BullTerrier04	Bull Terrier
BullTerrier05	Bull Terrier
Bulldog01	Bulldog
CE073	Cairn Terrier
CFA.105990	English Setter
CFA.107833	Field Spaniel
CFA.107834	Standard Schnauzer
CFA.107835	Dachshund
CFA.107836	Bouvier des Flandres
CFA.107837	Great Dane
CFA.107838	Border Terrier
CFA.107841	Greater Swiss Mountain Dog
CFA.107842	Standard Poodle
CFA.109668	Dachshund
CFA.109669	Spinone Italiano
CFA.109670	Berger Picard
CFA.109672	Gordon Setter
CFA.117995	Beagle
CFA.117996	English Setter
CFA.117997	Great Dane
CFA.117998	Irish Terrier
CFA.118001	Standard Schnauzer
CFA.118002	Standard Schnauzer

CK006	Cavalier King Charles Spaniel
CK023	Cavalier King Charles Spaniel
CP003	English Cocker Spaniel
CR039	Curly-Coated Retriever
CaneCorso01	Cane Corso
Chinook01	Chinook
ClumberSpaniel01	Clumber Spaniel
CockerSpanielEnglish01	English Cocker Spaniel
CockerSpanielEnglish03	English Cocker Spaniel
CockerSpanielEnglish04	English Cocker Spaniel
CockerSpanielEnglish05	English Cocker Spaniel
DD116	Great Dane
BullTerrier06	Bull Terrier
DH0117	Dachshund
DH126	Dachshund
DO242	Doberman Pinscher
DS043	German Shepherd Dog
DS051	German Shepherd Dog
Dachshund01	Dachshund
Doberman01	Doberman Pinscher
Doberman02	Doberman Pinscher
Doberman03	Doberman Pinscher
Doberman04	Doberman Pinscher
Elo01	Elo
EnglishMastiff01	Mastiff (English)
EnglishMastiff02	Mastiff (English)
EnglishPointer01	Pointer (English)
EnglishPointer02	Pointer (English)
EnglishSetter01	English Setter
EnglishSpringerSpaniel01	English Springer Spaniel
EnglishSpringerSpaniel03	English Springer Spaniel
EntlebucherSennenhund01	Entlebucher Sennenhund
EntlebucherSennenhund02	Entlebucher Sennenhund
EntlebucherSennenhund06	Entlebucher Sennenhund
EntlebucherSennenhund07	Entlebucher Sennenhund
EntlebucherSennenhund08	Entlebucher Sennenhund
FB065	French Bulldog
FinnishLapphund01	Finnish Lapphund
FlatcoatedRetriever01	Flat-Coated Retriever
FlatcoatedRetriever02	Flat-Coated Retriever
FlatcoatedRetriever03	Flat-Coated Retriever
FonniDog01	Fonni's Dog
FrenchBulldog01	French Bulldog
GS104	Gross spitz

GermanShepherd04	German Shepherd Dog
GermanShepherd05	German Shepherd Dog
GermanShepherd12	German Shepherd Dog
GermanWirehairedPointer01	German Wirehaired Pointer
GoldenRetriever08	Golden Retriever
GordonSetter01	Gordon Setter
GreatDane01	Great Dane
GreatDane02	Great Dane
GreatPyrenees01	Great Pyrenees
GreaterSwissMountainDog01	Greater Swiss Mountain Dog
Greyhound03	Greyhound
Greyhound05	Greyhound
Greyhound06	Greyhound
Greyhound07	Greyhound
Greyhound08	Greyhound
HW1706	Hovawart
Helsinki_BC1029	Border Collie
IrishSetter01	Irish Setter
IrishTerrier01	Irish Terrier
IrishWaterSpaniel01	Irish Water Spaniel
IrishWaterSpaniel02	Irish Water Spaniel
IrishWaterSpaniel03	Irish Water Spaniel
IrishWolfhound01	Irish Wolfhound
ItalianGreyhound01	Italian Greyhound
ItalianGreyhound02	Italian Greyhound
JT007	German Hunting Terrier
JackRussellTerrier02	Jack Russell Terrier
JackRussellTerrier03	Jack Russell Terrier
JackRussellTerrier04	Jack Russell Terrier
JackRussellTerrier05	Jack Russell Terrier
Jamthund01	Jamthund
Keeshond01	Keeshond
KerryBlueTerrier02	Kerry Blue Terrier
Komondor01	Komondor
Kromfohrlander01	Kromfohrlander
LR1030	Lagotto Romagnolo
LabradorRetriever04	Labrador Retriever
LabradorRetriever07	Labrador Retriever
LagottoRomagnolo01	Lagotto Romagnolo
LagottoRomagnolo02	Lagotto Romagnolo
LagottoRomagnolo03	Lagotto Romagnolo
LagottoRomagnolo04	Lagotto Romagnolo
Landseer01	Landseer
LapponianHerder01	Lapponian Herder

Leonberger01	Leonberger
Lowchen01	Lowchen
MA0163	Belgian Malinois
MA142	Belgian Malinois
MS04563	Miniature Schnauzer
MS04593	Miniature Schnauzer
MiniaturePoodle01	Miniature Poodle
MiniatureSchnauzer01	Miniature Schnauzer
NW062	Norwich Terrier
NW152	Norwich Terrier
NW206	Norwich Terrier
NW255	Norwich Terrier
NorwegianElkhound01	Norwegian Elkhound
NorwegianElkhound02	Norwegian Elkhound
NorwegianLundehund01	Norwegian Lundehund
NorwegianLundehund02	Norwegian Lundehund
NorwegianLundehund03	Norwegian Lundehund
PER00075	Yorkshire Terrier
PER00204	Yorkshire Terrier
PER00409	Yorkshire Terrier
PL116	Poodle unspecified variety
PT49	Portugal Village Dog
PembrokeWelshCorgi01	Pembroke Welsh Corgi
PembrokeWelshCorgi02	Pembroke Welsh Corgi
PembrokeWelshCorgi03	Pembroke Welsh Corgi
PetitBassetGriffonVendeen01	Petit Basset Griffon Vendeen
Pomeranian01	Pomeranian
PortuguesePodengo01	Portuguese Podengo
PortugueseWaterDog01	Portuguese Water Dog
PortugueseWaterDog02	Portuguese Water Dog
PortugueseWaterDog04	Portuguese Water Dog
PortugueseWaterDog09	Portuguese Water Dog
PortugueseWaterDog11	Portuguese Water Dog
Rottweiler01	Rottweiler
Rottweiler03	Rottweiler
Rottweiler04	Rottweiler
SS004	Shetland Sheepdog
SaintBernard01	Saint Bernard
SaintBernard02	Saint Bernard
ScottishDeerhound01	Scottish Deerhound
ScottishTerrier01	Scottish Terrier
ScottishTerrier02	Scottish Terrier
ScottishTerrier03	Scottish Terrier
ScottishTerrier04	Scottish Terrier

	ShetlandSheepdog01	Shetland Sheepdog
	ShetlandSheepdog02	Shetland Sheepdog
	SoftCoatedWheatenTerrier01	Soft Coated Wheaten Terrier
	SoftCoatedWheatenTerrier02	Soft Coated Wheaten Terrier
	SoftCoatedWheatenTerrier03	Soft Coated Wheaten Terrier
	SoftCoatedWheatenTerrier04	Soft Coated Wheaten Terrier
	SpanishGalgo01	Spanish Galgo
	SpanishWaterDog01	Spanish Water Dog
	StandardPoodle01	Standard Poodle
	StandardPoodle02	Standard Poodle
	StandardPoodle03	Standard Poodle
	StandardSchnauzer01	Standard Schnauzer
	SwedishLapphund01	Swedish Lapphund
	TA001	Airedale Terrier
	ToyPoodle01	Toy Poodle
	VillDog_Portugal01	Portugal Village Dog
	VillDog_Portugal02	Portugal Village Dog
	Vizsla01	Vizsla
	WE006	Weimaraner
	WS42321902_S16	Rottweiler
	WW558	West Highland White Terrier
	WestHighlandWhiteTerr02	West Highland White Terrier
	WestHighlandWhiteTerr04	West Highland White Terrier
	WestHighlandWhiteTerr05	West Highland White Terrier
	WestHighlandWhiteTerr06	West Highland White Terrier
	Whippet01	Whippet
Southern East Asian indigenous dogs (SEAID)	ChongqingDog01	
	IndigenousDogVietnam01	
	IndigenousDogVietnam02	
	IndigenousDogVietnam03	
	IndigenousDogVietnam04	
	IndigenousDogVietnam05	
	QingchuanDog01	
	VillDog_China02	
	VillDog_China08	
	VillDog_China09	
	VillDog_China20	
	VillDog_China21	
	VillDog_China22	
	VillDog_China23	
	VillDog_China24	
	VillDog_China25	
	VillDog_China26	
	VillDog_China27	

	VillDog_China28
	VillDog_China29
	VillDog_China30
	VillDog_China31
	VillDog_China32
	VillDog_China33
	VillDog_China34
	VillDog_China35
	VillDog_China36
	VillDog_China37
	VillDog_China38
	VillDog_China39
	VillDog_China51
	VillDog_Taiwan01
	VillDog_Vietnam01
	VillDog_Vietnam02
	VillDog_Vietnam03
	VillDog_Vietnam04
	VillDog_Vietnam05
	VillDog_Vietnam06
Grey Wolves	AlaskanWolf
	WO001_895
	WO002_732
	WO003_636
	Wolf01
	Wolf02
	Wolf03
	Wolf04
	Wolf05
	Wolf06
	Wolf07
	Wolf08
	Wolf19
	Wolf20
	Wolf21
	Wolf22
	Wolf23
	Wolf24
	Wolf27
	Wolf28
	Wolf29
	Wolf30
	Wolf31
	Wolf32

	Wolf33	
	Wolf34	
	Wolf35	
	Wolf36	
	Wolf37	
	Wolf38	
	Wolf39	
	Wolf41	
	Wolf42	
	WolfTibetan01	
	WolfTibetan02	
	WolfTibetan03	
	WolfTibetan04	
	WolfTibetan05	
	WolfTibetan06	
	WolfTibetan07	
	WolfTibetan08	
	TibetanMastiff01	Tibetan Mastiff
	TibetanMastiff02	Tibetan Mastiff
	TibetanMastiff03	Tibetan Mastiff
	TibetanMastiff04	Tibetan Mastiff
	TibetanMastiff05	Tibetan Mastiff
	TibetanMastiff06	Tibetan Mastiff
	TibetanMastiff07	Tibetan Mastiff
	TibetanMastiff08	Tibetan Mastiff
	TibetanMastiff09	Tibetan Mastiff
	TibetanMastiff10	Tibetan Mastiff
	TibetanMastiff11	Tibetan Mastiff
	VillDog_China07	
Tibetan dogs	VillDog_China40	
(TD)	VillDog_China41	
	VillDog_China42	
	VillDog_China43	
	VillDog_China44	
	VillDog_China45	
	VillDog_China46	
	VillDog_China47	
	VillDog_China48	
	VillDog_China49	
	VillDog_China50#	
	VillDog_China52#	
	TibetanTerrier01#	Tibetan Terrier
	TibetanTerrier02#	Tibetan Terrier

#: outliers in the result of PCA.

Table S3. The local ancestral inference for Tibetan dogs with PCAdmix.

Sample ID of Tibetan dogs	Proportion from European breeds	Proportion from SEAID	Proportion unclassified#
TibetanMastiff01	28.93%	57.91%	13.15%
TibetanMastiff02	29.65%	57.77%	12.58%
TibetanMastiff03	32.05%	54.50%	13.45%
TibetanMastiff04	31.95%	54.56%	31.95%
TibetanMastiff05	32.46%	54.04%	13.50%
TibetanMastiff06	31.97%	54.59%	13.44%
TibetanMastiff07	33.00%	53.21%	13.78%
TibetanMastiff08	27.03%	59.88%	13.09%
TibetanMastiff09	32.00%	54.46%	13.55%
TibetanMastiff10	34.25%	52.99%	12.75%
TibetanMastiff11	32.97%	53.51%	13.52%
VillDog_China07	40.79%	46.31%	12.90%
VillDog_China40	28.31%	59.08%	12.61%
VillDog_China41	29.37%	58.19%	12.44%
VillDog_China42	31.23%	57.43%	11.34%
VillDog_China43	28.92%	58.67%	12.42%
VillDog_China44	27.76%	59.78%	12.46%
VillDog_China45	31.42%	56.23%	12.34%
VillDog_China46	29.87%	57.67%	12.46%
VillDog_China47	27.20%	59.91%	12.89%
VillDog_China48	29.34%	58.80%	11.87%
VillDog_China49	30.54%	57.57%	11.89%
TibetanTerrier01	82.08%	12.01%	5.91%
TibetanTerrier02	80.03%	13.76%	6.21%
VillDog_China50	94.98%	2.24%	2.78%
VillDog_China52	68.77%	21.01%	10.21%

#: haplotypes are assigned as “unclassified” if the confidence is less than 0.9. Four Tibetan dogs defined as outliers are noted in red.

Table S5. Lactose tolerance test and lactase persistence allele genotyping for 32 adult Tibetans.

**Sample ID**	**DNA ID**	***-13838G>A* genotyping**	**Sex**	**Age**	**Hydrogen breath test (increment ppm value within 3 h)**	**Phenotype**
DXT943	LCTT1	GA	Male	30	17	lactase persistence
DXT944	LCTT2	GG	Female	23	121	lactase non-persistence
DXT945	LCTT3	GG	Female	29	43	lactase non-persistence
DXT946	LCTT4	GG	Female	29	79	lactase non-persistence
DXT947	LCTT5	GG	Female	34	98	lactase non-persistence
DXT948	LCTT6	GG	Male	40	26	lactase non-persistence
DXT949	LCTT7	GG	Male	34	99	lactase non-persistence
DXT950	LCTT8	GG	Female	63	49	lactase non-persistence
DXT951	LCTT9	GA	Male	33	52	lactase non-persistence
DXT952	LCTT10	GG	Male	24	126	lactase non-persistence
DXT953	LCTT11	GG	Female	30	26	lactase non-persistence
DXT954	LCTT12	GG	Female	22	114	lactase non-persistence
DXT955	LCTT13	GG	Male	39	199	lactase non-persistence
DXT957	LCTT15	GG	Male	52	102	lactase non-persistence
DXT959	LCTT17	GG	Female	37	77	lactase non-persistence
DXT960	LCTT18	GG	Male	37	121	lactase non-persistence
DXT961	LCTT19	GG	Female	23	137	lactase non-persistence
DXT962	LCTT20	GG	Male	38	160	lactase non-persistence
DXT963	LCTT21	GG	Male	31	78	lactase non-persistence
DXT966	LCTT24	GG	Female	17	93	lactase non-persistence
DXT967	LCTT25	GG	Female	20	50	lactase non-persistence
DXT968	LCTT26	GG	Female	23	93	lactase non-persistence
DXT969	LCTT27	GG	Female	27	62	lactase non-persistence
DXT970	LCTT28	GG	Male	53	26	lactase non-persistence
DXT971	LCTT29	GG	Female	47	60	lactase non-persistence
DXT972	LCTT30	GG	Female	32	117	lactase non-persistence
DXT973	LCTT31	GG	Male	60	82	lactase non-persistence
DXT974	LCTT32	GG	Male	37	86	lactase non-persistence
DXT975	LCTT33	GG	Female	22	31	lactase non-persistence
DXT976	LCTT34	GG	Female	43	51	lactase non-persistence
DXT977	LCTT35	GG	Female	30	6	lactase persistence
DXT978	LCTT36	GG	Female	37	78	lactase non-persistence

Table S6. Information of double-stranded oligonucleotide probes in EMSAs.

Probe	Sequence（5'— 3'）	Length (bp)	Note
-13838*G-F	ATTTTTAGATTGTTCTTTGAGCCCTGCATTCCACGAGGAT	40	5'-biotin
-13838*G-R	ATCCTCGTGGAATGCAGGGCTCAAAGAACAATCTAAAAAT	40	5'-biotin
-13838*A-F	ATTTTTAGATTGTTCTTTGAACCCTGCATTCCACGAGGAT	40	5'-biotin
-13838*A-R	ATCCTCGTGGAATGCAGGGTTCAAAGAACAATCTAAAAAT	40	5'-biotin
-13838*G-F-Competitor	ATTTTTAGATTGTTCTTTGAGCCCTGCATTCCACGAGGAT	40	Unlabeled
-13838*G-R- Competitor	ATCCTCGTGGAATGCAGGGCTCAAAGAACAATCTAAAAAT	40	Unlabeled
-13838*A-F- Competitor	ATTTTTAGATTGTTCTTTGAACCCTGCATTCCACGAGGAT	40	Unlabeled
-13838*A-R- Competitor	ATCCTCGTGGAATGCAGGGTTCAAAGAACAATCTAAAAAT	40	Unlabeled
Mut-F-Unspecific-Competitor	ATTTTTAGATTGTTCTTCAGGTTCTGCATTCCACGAGGAT	40	Unlabeled
Mut-R-Unspecific-Competitor	ATCCTCGTGGAATGCAGAACCTGAAGAACAATCTAAAAAT	40	Unlabeled

Note:

For the unlabeled competitors, the central 8 bp surrounding the consensus HNF4A binding site were mutated.

Fig. S1. Principal component analysis showing genetic affiliation among Tibetan dogs, southern East Asian indigenous dogs, European breeds, and wolves.

Most Tibetan dogs (TD) cluster together with southern East Asian indigenous dogs (SEAID). Four Tibetan dogs clustering together with European breeds (EB) are defined as outliers.

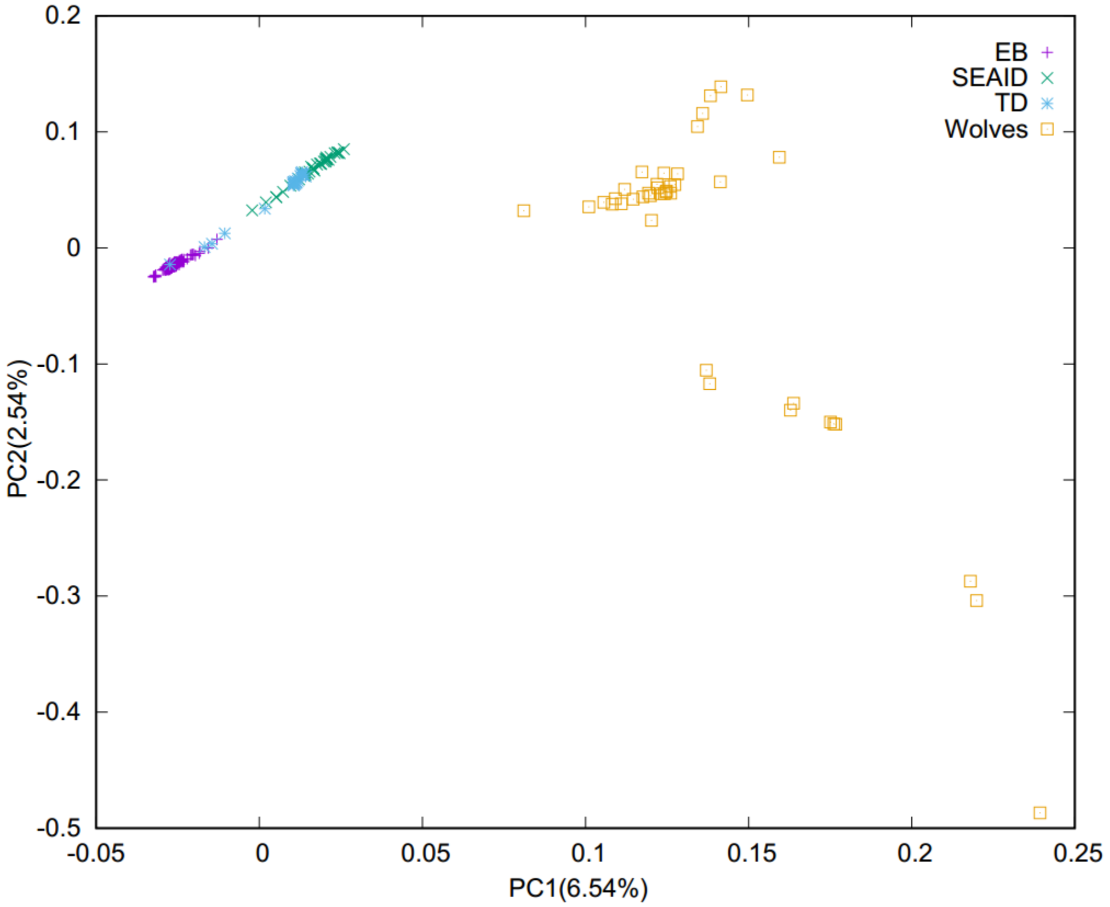

Fig. S2. ADMIXTURE analysis for Tibetan dogs, southern East Asian indigenous dogs, European breeds, and wolves.

The result is shown when K = 3 with the lowest cross-validation error. Most Tibetan dogs (TD) and southern East Asian indigenous dogs (SEAID) share dominant genetic component (green). Four Tibetan dogs have dominant genetic component (blue) prevalent in European breeds (EB) are defined as outliers.

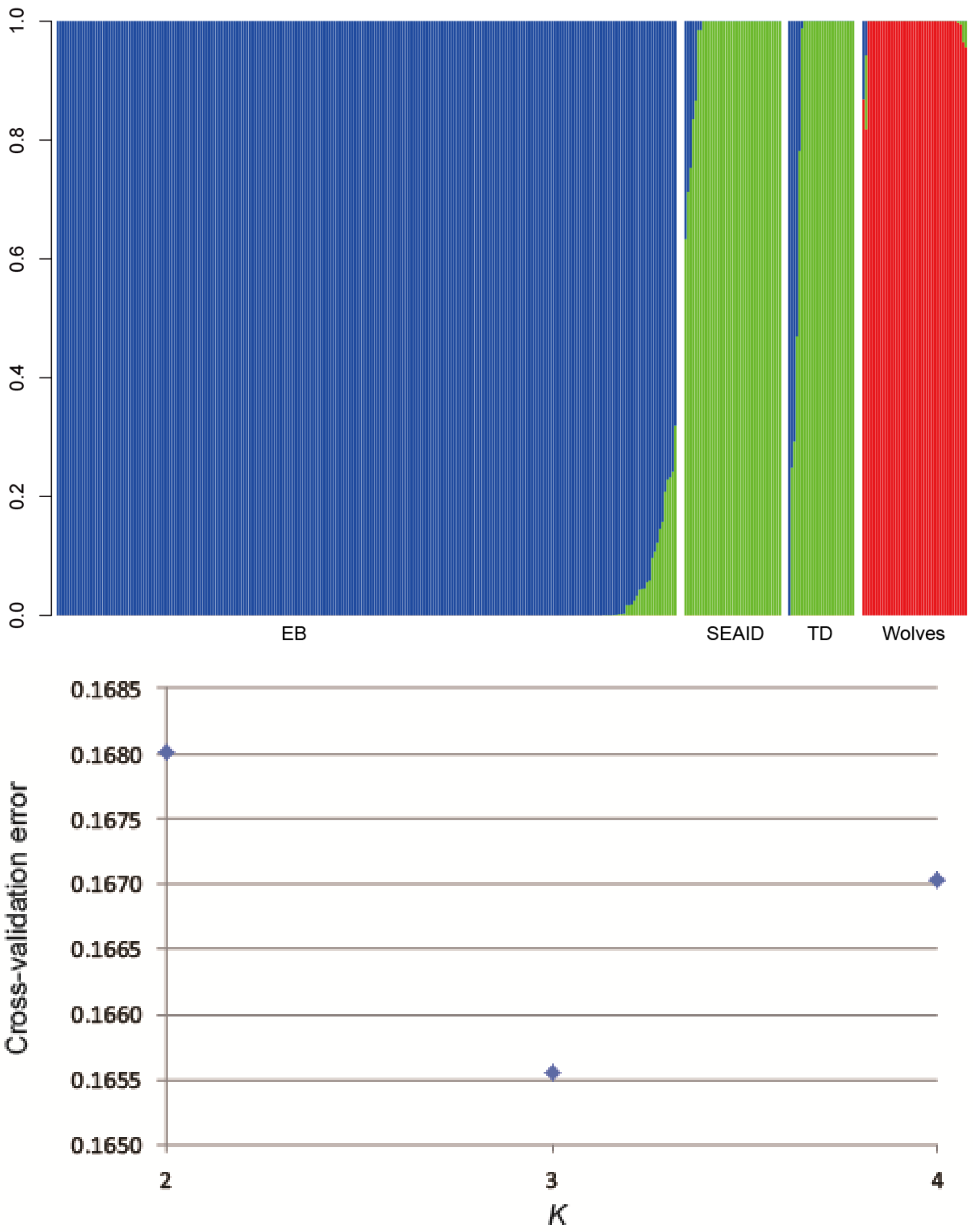

Fig. S3. Model selection by momi2.

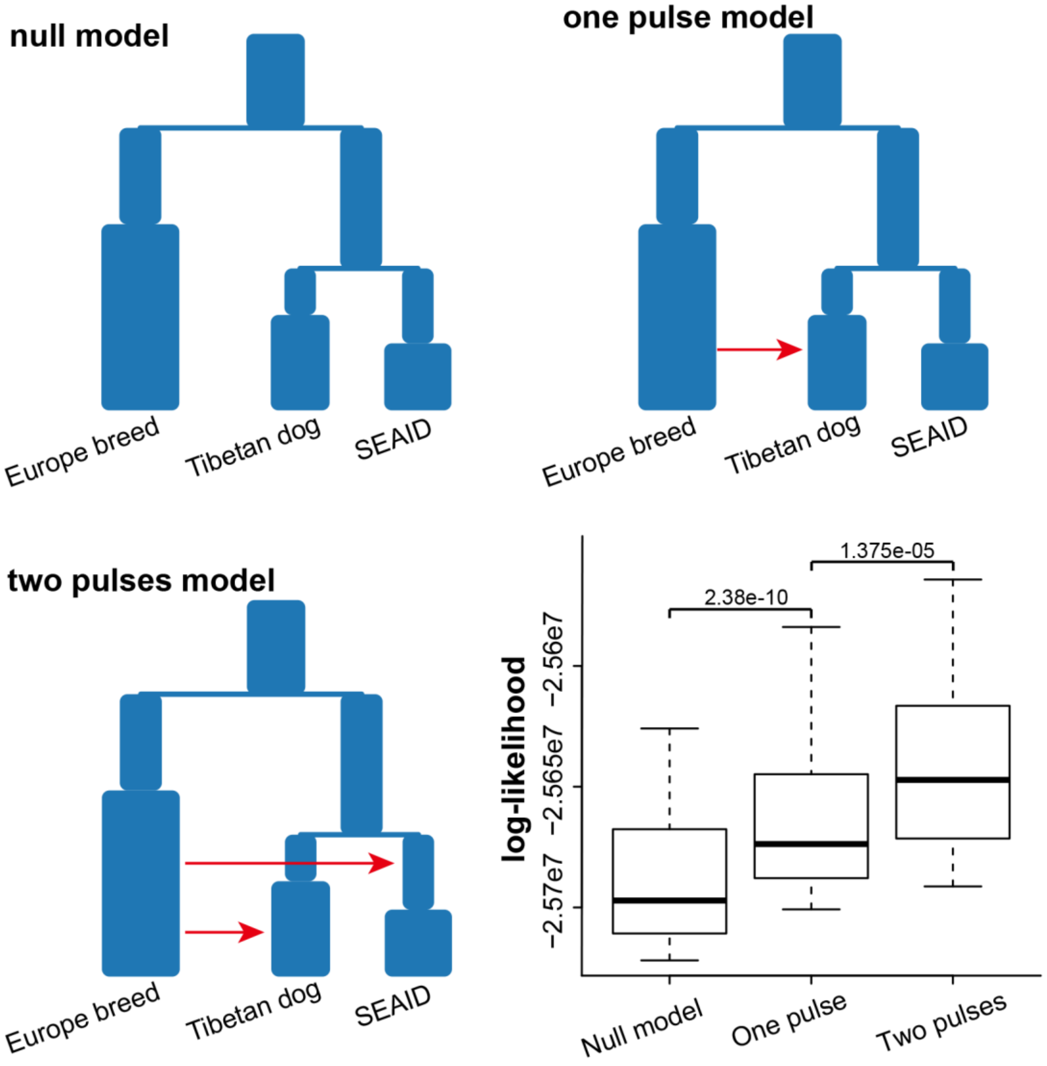

Fig. S4. The selected model with two pulses of gene flow and parameter estimation.

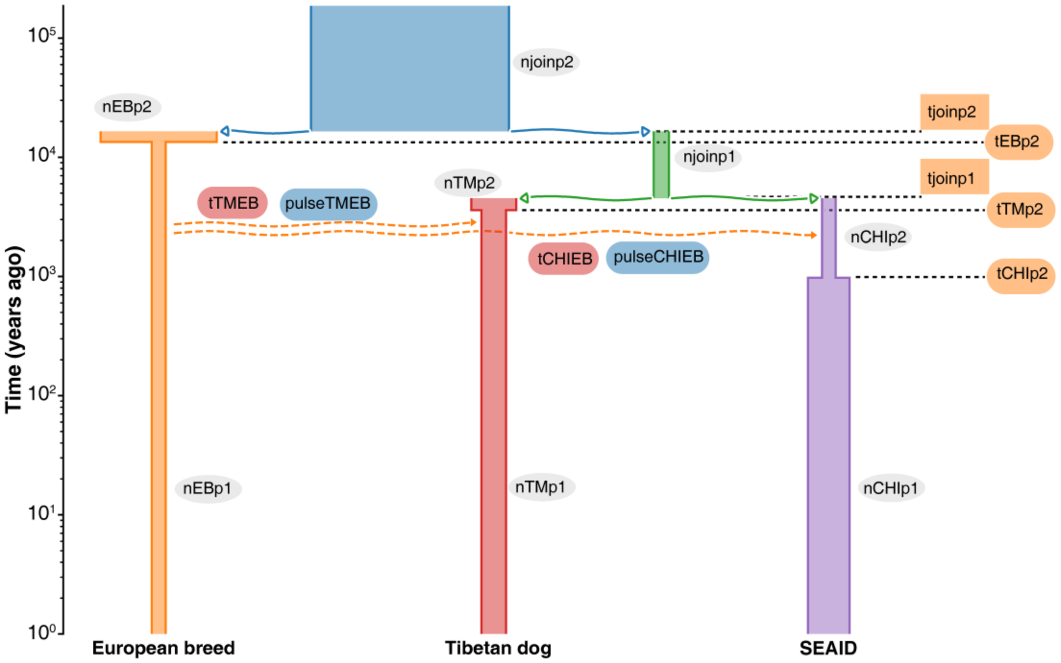

Parameters	Description	Estimation	Lower 95% CI	Upper 95% CI
njoinp2	Ne common ancestor of European and Asian dog s	81,573	79,643	82,441
njoinp1	Ne common ancestor of Asian dog	6,295	5,709	12,126
nEBp2	Ne European dog before Ne change	47,875	5,164	64,415
nEBp1	Ne European dog after Ne change	5,778	4,401	10,552
nTMp2	Ne Tibetan dog before Ne change	18,481	6,848	79,469
nTMp1	Ne Tibetan dog after Ne change	10,097	4,228	14,811
nCHIp2	Ne Southeast Asian village dog before Ne change	5,635	3,229	44,340
nCHIp1	Ne Southeast Asia n village dog after Ne change	17,146	3,592	42,090
tjoinp2	Time of split for European dog from common ancestor of European and Asian dogs	16,499	11,849	29,198
tjoinp1	Time of split for Tibetan dog from common ancestor of Asian dog	4,523	2,805	8,530
tEBp2	Time of Ne change for European dog	13,438	1,593	23,163
tTMp2	Time of Ne change for Tibetan dog	3,602	222	5,426
tCHIp2	Time of Ne change for Southeast Asian village dog	976	122	6,021
tTMEB	Time of gene flow from European dog to Tibetan dog	3,595	1,320	5,257
pulseTMEB	Proportion for gene flow from European dog to Tibetan dog	0.3900	0.2583	0.3900
tCHIEB	Time of gene flow from European dog to Southeast Asian dog	3,687	591	5,211
pulseCHIEB	Proportion for gene flow from European dog to Southeast Asian dog	0.3000	0.1561	0.3000

Fig. S5. The admixture history of Tibetan dogs inferred by MSMC-IM.

Although only one peak of gene flow occurred between European breeds (EB) and Tibetan dogs (TD), one peak of gene flow was detected between southern East Asian indigenous dogs (SEAID) and European breeds almost at the same time. The haplotypes of dogs sequenced with the highest depth were used. One Tibetan indigenous dog (DQ28) and one Tibetan Mastiff (DQZA81) with the highest coverage are used for the representative of Tibetan dogs. For European breeds, we choose one individual with the highest coverage from two breeds (BullTerrier06 and Greyhound06), respectively.

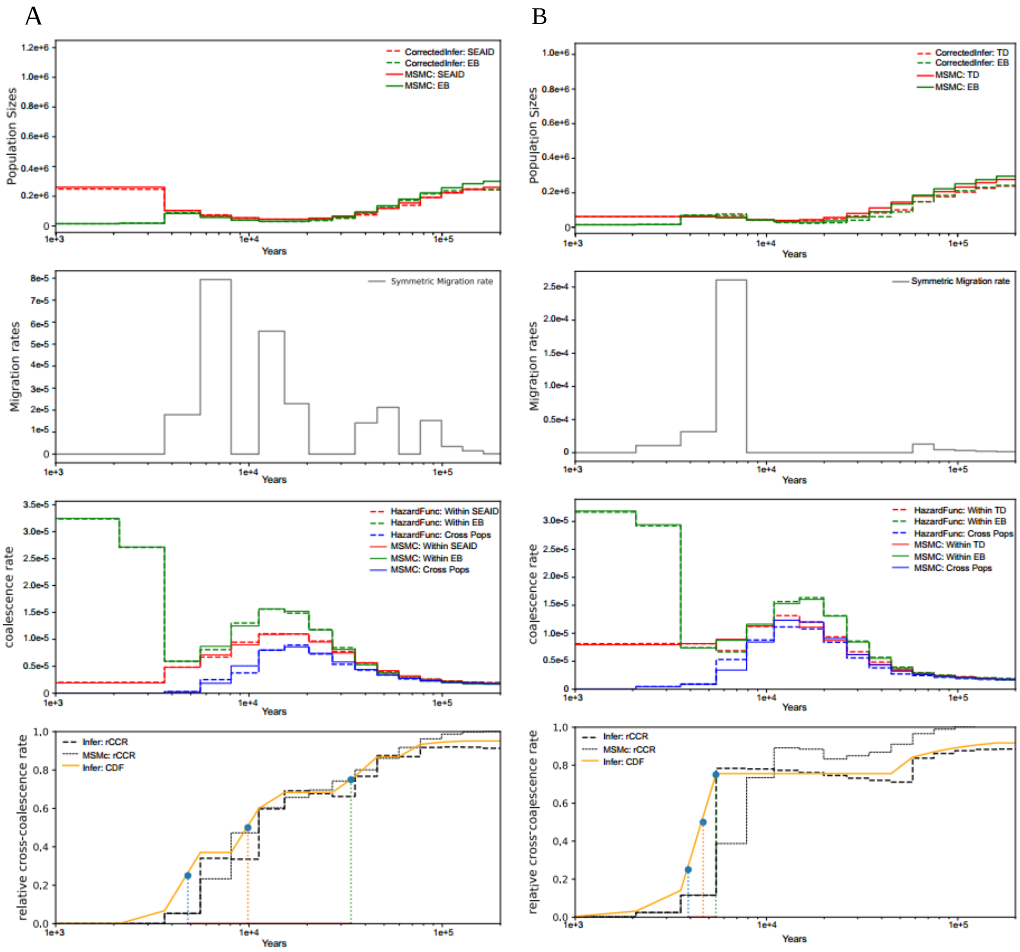

Fig. S6. Sliding-window *D* and *fdM* statistics (outgroup-Andean fox, European breeds; Tibetan dogs, southern East Asian indigenous dogs) around the dog lactase gene.

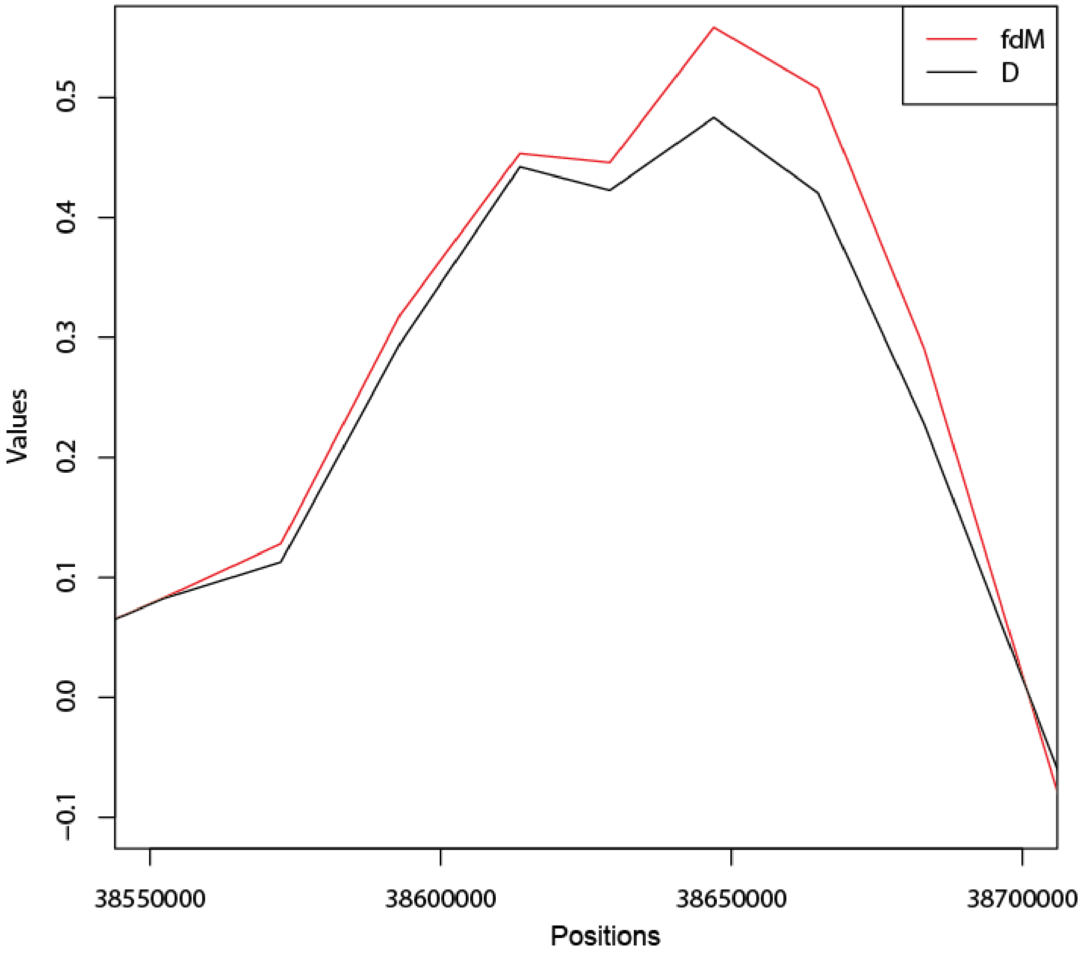

Supplement: Supplementary file 2 — Additional file 2: Table S2. Sample information for genomic data of dogs and wolves. Table S3. The local ancestral inference for Tibetan dogs with PCAdmix. Table S5. Lactose tolerance test and lactase persistence allele genotyping for 32 adult Tibetans. Table S6. Information of double-stranded oligonucleotide probes in EMSAs. Fig. S1. Principal component analysis showing genetic affiliation among Tibetan dogs, southern East Asian indigenous dogs, European breeds, and wolves. Fig. S2. ADMIXTURE analysis for Tibetan dogs, southern East Asian indigenous dogs, European breeds, and wolves. Fig. S3. Model selection by momi2. Fig. S4. The selected model with two pulses of gene flow and parameter estimation. Fig. S5. The admixture history of Tibetan dogs inferred by MSMC-IM. Fig. S6. Sliding window D and fdM statistics (outgroup Andean fox, European breeds; Tibetan dogs, southern East Asian indigenous dogs) around the dog lactase gene. [file 12915_2023_1707_MOESM2_ESM.pdf]
